# Association between activity energy expenditure and peak oxygen consumption with sarcopenia

**DOI:** 10.1186/s12877-018-0993-y

**Published:** 2018-12-03

**Authors:** Daniel Bunout, Gladys Barrera, Sandra Hirsch, Teresa Jimenez, María Pia de la Maza

**Affiliations:** Institute of Nutrition and Food Technology, University of Chile, PO Box 138-11, Santiago, Chile

**Keywords:** Sarcopenia, Sedentariness, Peak oxygen consumption, Actigraphy

## Abstract

**Background:**

Sedentariness may be an important risk factor for sarcopenia. The aim of this work was to assess the association between muscle mass and strength and markers of usual physical activity such as activity energy expenditure and peak oxygen uptake.

**Methods:**

Young and old participants were assessed measuring body composition by DEXA (double beam X ray absorptiometry), handgrip strength, peak oxygen consumption and workload during an exercise calorimetry in a braked cycle ergometer and a 72 h activity energy expenditure using Actiheart actigraphs. A heart rate/energy expenditure curve derived from the exercise calorimetry was used to calibrate each actigraph. Sarcopenia was defined as having an appendicular fat free mass index below 7.5 kg/m^2^ and 5.6 kg/m^2^ in men and women respectively, or a handgrip strength z score below 1, using local normal data or having both parameters below the cutoff points.

**Results:**

We analyzed data from 192 assessments performed in participants aged 22 to 88 years (106 women). Sarcopenic participants (as determined by muscle mass, strength or both) had a significantly lower peak oxygen uptake and work load and a significantly lower activity energy expenditure. When analyzing lean mass and strength as continuous variables, peak oxygen consumption was a significant predictor of fat free mass in men. Among women, the association was observed only when percentage of muscle mass was expressed as a z score.

**Conclusions:**

Activity energy expenditure and peak oxygen consumption are associated with a lower muscle mass and the presence of sarcopenia and should be considered as risk factors for this condition.

## Background

Sarcopenia or the loss of muscle mass and function may occur in any moment of life, but old age is a risk factor and a condition in which its functional consequences are more striking. There are many pathogenic hypotheses to explain the loss of muscle mass. Among these, sedentariness is always mentioned, but its real association with sarcopenia both in young and old people has not been fully tested.

If sedentariness has a pathogenic role on sarcopenia, this factor should be equally important in young and old people (defined as subjects aged 60 years or more), and there should be an association between usual physical activity and loss of muscle mass. There are previous studies looking for this association. Park et al. found an association between year-long physical activity measured using pedometers and muscle mass in older people [1]. A further study by the same authors following older people for five years showed that the loss muscle mass in the study period was also associated with sedentariness determined using accelerometry [2]. There are several cross sectional studies that confirm this association [3].

An accurate method to measure usual physical activity is to determine activity energy expenditure. Doubly labeled water is the gold standard, however it is extremely expensive. An alternative is the use of actigraphy, whose accuracy increases when heart rate is also measured and is calibrated with individual heart rate/activity curves. There is a good concordance between doubly labeled water measurements and actigraphy with heart rate determination [4]. However some authors suggest that wearable devices lack accuracy [5].

The measurement of peak oxygen consumption by indirect calorimetry gives a precise estimation of the aerobic capacity of an individual. It is proportional to the usual physical activity and a direct indicator of fitness. Although it is influenced by age and genetic factors, usual physical activity is one of its main determinants [6]. Previous reports showed a direct association between muscle mass and peak oxygen consumption, speculating that a lower amount of metabolically active tissue would explain this association. However it is also possible to asseverate that a lower fitness derived from sedentariness, causes a reduction in muscle mass [7]. Therefore this parameter should provide an objective measurement that is inversely associated with long term sedentariness.

## Methods

With the hypothesis that sedentariness is a main determinant of sarcopenia, the aim of this study was to evaluate whether daily physical activity and maximum oxygen consumption are determinants of muscle mass and function, regardless of age.

We analyzed data obtained from healthy volunteers participating in several studies on body composition, exercise capacity and aging. When the participants signed the informed consent to participate in the aforementioned studies, they allowed us to use their data in secondary analyses. Subjects living in the community aged 22 to 88 years were included. For all studies, we excluded participants with chronic debilitating diseases such as cancer, renal or cardiac failure or chronic infections. Individuals who could not reach the clinic by their own means, were unable to understand and sign the consent form or those who admitted abnormal consumption of alcohol or illicit drugs, also were excluded.

All participants had the following assessments performed and recorded:

### Clinical assessment

Clinical history and physical examination including measurement of blood pressure, weight, height, waist circumference and a questionnaire about usual physical activity (International Physical Activity Questionnaire or IPAQ) [8].

### Handgrip strength measurement

Handgrip strength was measured using a Therapeutic Instruments (Clifton NJ, United State) dynamometer. Three measurements were made in each hand and the higher value obtained for either hand was recorded. The measurement error of this parameter in repeated assessments in the same individual was 8.7%.

### Body composition assessment

Measurement of body composition by double beam X ray absorptiometry (DEXA) using a Lunar iDEXA equipment. Subjects were fasting at the moment of the assessment and had a normal fluid intake the previous day. A normal hydration status was confirmed clinically, looking for the absence of dehydration signs, peripheral edema or orthostatic hypotension. GE (General Electric) software version 13.6 was used. The measurement error of the method for body composition is 2.9% in our hands [9].

### Indirect calorimetry

Submaximal oxygen consumption after an incremental test in a braked cycle ergometer was measured using a Sensormedics Vmax Encore 29 equipment. The incremental exercise test was started at a 10-watt ramp with 10-watt increases per minute or a 15-watt ramp with 15-watt increases (depending on age and the self-reported physical condition of participants) until volitional exhaustion, with a cadence of 60 rpm. The test was stopped if the participant could not maintain the cadence after the stage change or if the respiratory exchange ratio exceeded 1.3 [10]. A facemask was used to collect respiratory gases and the breath by breath method was used to measure oxygen consumption and CO_2_ production. Peak oxygen consumption was determined at the moment in which the participant was not able to maintain the cycling cadence. Gross work efficiency was calculated as the ratio between work rate and energy expended in joules multiplied by 100, at submaximal work rates [11]. To test the reproducibility of the measurement, we repeated the test within one month in 20 participants. The rho for concordance of two peak oxygen consumption measurements carried out in the same individual was 0.8. The mean peak oxygen consumption values in the first and second measurements were 20.7 ± 6 and 20.8 ± 7 ml/min/kg.

#### Seventy two hours actigraphy

An actigraph (Actiheart®) was installed to all participants for 72 h during weekdays for actigraphy and heart rate measurement. The devices were individually calibrated with the heart rate/energy expenditure curve obtained during the exercise calorimetry. The individual correlation between energy expenditure and heart rate in participants was 0.88 ± 0.07. Total energy expenditure (TEE), activity energy expenditure (AEE) and physical activity level (PAL) were determined (Actiheart software version 4.0.32, www.camntech.com).

### Data analysis and statistical methods

#### Definition of sarcopenia

Using body composition data obtained by DEXA, appendicular fat free mass index was calculated dividing appendicular fat free mass/height(m)^2^. The cutoff point for this parameter used to determine sarcopenia was a value below 7.5 kg/m^2^ in men and 5.6 kg/m^2^ in women as we have recently proposed [12], following a recommendation made by a Brazilian group of researchers [13]. Appendicular fat free mass and fat free mass index were also expressed as z scores, calculated for decade of age (from 20 to 90 years) and sex, using the normal body composition data from our laboratory [11]. Skeletal muscle mass was also estimated using the equation proposed by Kim et al. [14] as [1.13 x appendicular fat free mass] - [0.02 x age] + [0.61 x sex] 165 + 0.97 (sex: female = 0, male = 1). The percentage of skeletal muscle mass was then calculated as appendicular fat free mass* 100/skeletal muscle mass.

Handgrip strength was transformed to sex and age-specific z scores and a value below 1 was defined as indicative of sarcopenia. To obtain normal values for hand grip strength, we used measurements performed in 366 healthy subjects aged 20 to 89 years (255 females), as previously reported [15]. The mean value for each parameter and its standard deviation were calculated for each gender in ten year intervals from 20 years of age to over 80 years.

We classified participants as sarcopenic using three criteria: having an appendicular fat free mass index or a handgrip strength below their cutoff points or having both parameters below their cutoff points. We compared sarcopenic and non-sarcopenic participants, using these three different criteria. We also treated body composition and handgrip strength data as continuous variables and included them in multiple regression models. In these models, the independent variables added were age, peak oxygen uptake, gross work efficiency (as described earlier), physical activity level derived from actigraphy and total physical activity derived from IPAQ questionnaire. We were especially careful to avoid including independent variables that were highly correlated, to avoid biases in the regression model.

For analysis purposes, participants were divided according to age, in young (< 60 years) and old (> = 60 years) groups. This is the age used to define older people in Chile. All subsequent comparisons and regression models were performed separately for men and women, considering the striking gender differences in muscle strength and mass. Normality of variable distribution was assessed using the Shapiro Wilk test. Variables with a normal distribution are expressed as mean ± standard deviation and otherwise as median (interquartile range). Parametric statistical tests such as t test were used to compare normally distributed variables. Otherwise, we used non-parametric tests such as Kruskal Wallis anova. For the multiple regression models a p of 0.1 was used as cutoff to accept or remove variables from the model.

## Results

Data from 192 assessments performed in participants aged from to 22 to 88 years (106 women) were analyzed. Table 1 shows clinical, body composition, exercise calorimetry and actigraphy features of participants, divided by gender and age range. Women and older people had lower fat free mass, handgrip strength, peak oxygen consumption and workload and activity energy expenditure. Young women reported a total physical activity in the physical activity questionnaire, which was significantly lower than the rest of the groups. The respiratory exchange ratio of 1.3 in all groups is an indication that real peak values were achieved during the exercise calorimetry. There was a significant correlation between peak oxygen uptake and activity energy expenditure measured by actigraphy, both in women and men (*r* = 0.53 and 0.37 respectively, *p* < 0.01). There was also a significant correlation between peak oxygen consumption and fat free mass in women and men (*r* = 032 and 0.62 respectively, p < 0.01). There was no association between total physical activity measured using the International Physical activity questionnaire and peak oxygen uptake or activity energy expenditure.

**Table 1:** Features of studied participants

	Young women (A) (*n* = 65)	Old women (B) (*n* = 41)	Young men (C) (*n* = 58)	Old men (D) (*n* = 28)	Significant differences
Demographic and clinical data					
Age (years)	36.2 ± 9.0^a^	70.9 ± 5.2	36.2 ± 7.7	69.1 ± 7.1	
Body mass index (kg/m^2^)	28.9 ± 1.8	29.0 ± 4.4	29.8 ± 2.0	27.5 ± 3.1	C vs D
Waist circumference (cm)	90.0 ± 5.7	99.6 ± 11.2	100.9 ± 6.0	98.8 ± 9.0	A vs B C D
Systolic blood pressure (mm Hg)	115.5 ± 10.7	128.5 ± 14.3	122.8 ± 9.6	131.8 ± 16.5	A vs C D C vs D
Diastolic blood pressure (mm Hg)	75.1 ± 8.7	71.5 ± 10.0	75.2 ± 7.7	82.1 ± 11.0	NS
Handgrip strength (kg)	25.0 ± 4.4	19.4 ± 4.1	42.3 ± 7.6	28.5 ± 5.6	All groups different
Handgrip strength (z score)	0.88 ± 1.60	− 0.35 ± 0.80	0.17 ± 1.57	−0.79 ± 1.18	A vs D C vs D
Body composition (DEXA)					
Total fat free mass (kg)	38.48 ± 2.92	35.46 ± 4.07	56.40 ± 5.96	47.97 ± 6.40	All groups different
Total fat mass (kg)	30.99 ± 3.76	30.24 ± 8.07	29.84 ± 4.76	24.04 ± 5.40	D vs A B C
Appendicular fat free mass (kg)	16.00 ± 1.52	14.10 ± 2.24	24.86 ± 2.97	19.61 ± 3.28	All groups different
Appendicular fat free mass index (kg/m2)	6.44 ± 0.50	6.04 ± 0.92	8.33 ± 0.84	7.14 ± 0.93	All groups different
Appendicular fat mass (kg)	13.30 ± 2.34	12.29 ± 3.42	10.51 ± 2.18	7.92 ± 1.78	A vs C D B vs C D C vs D
Percentage of skeletal muscle mass (%)	25.59 ± 1.88	22.96 ± 2.03	32.59 ± 2.39	29.53 ± 1.80	All groups different
Appendicular fat free mass (z score)	0.66 ± 0.80	0.43 ± 1.15	0.76 ± 0.90	− 0.02 ± 1.14	D vs A C
Percentage of skeletal muscle mass (z score)	−0.38 ± 0.70	−0.12 ± 0.70	−0.33 ± 0.79	−0.33 ± 0.51	D vs A
International physical activity questionnaire					
Total physical activity (MET-minutes/week)	630 (264–1252)^b^	1155 (531–2346)	1173 (626–1944)	1179 (544–2071)	A vs B C D
Exercise calorimetry					
Peak oxygen uptake (ml/min/kg)	17.6 ± 4.2	8.5 ± 2.3	25.6 ± 5.8	12.0 ± 3.2	All groups different
Peak respiratory exchange ratio (VCO_2_/VO_2_)	1.3 ± 0.1	1.3 ± 0.2	1.3 ± 0.2	1.3 ± 0.2	NS
Peak workload (Watt)	121.9 ± 30.5	41.0 ± 18.6	208.9 ± 50.3	66.1 ± 21.4	All groups different
Gross muscle efficiency (%)	26.0 ± 6.3	19.3 ± 7.8	24.4 ± 3.2	19.5 ± 4.2	D vs A C
Seventy two hours actigraphy					
Physical activity level (Total/Resting energy expenditure)	1.51 ± 0.23	1.39 ± 0.17	1.83 ± 0.41	1.59 ± 0.16	A vs C D B vs C D C vs D
Activity energy expenditure (Kcal/day)	530 (380–837)	287 (211–380)	1047 (679–1702)	653 (541–695)	A vs B C B vs C D C vs D

^a^= mean ± standard deviation ^b^ = median (interquartile range)

Table 2 compares men and women according to the presence of sarcopenia, defined using body composition, strength criteria or both. Peak oxygen consumption and work load and 72 h activity energy expenditure were lower in sarcopenic participants, whatever definition used. When sarcopenia was defined using only strength criteria, no differences in activity energy expenditure were observed between sarcopenic women and their non-sarcopenic counterparts.

**Table 2:** Features of participants with and without sarcopenia

	Women			Men		
	Without sarcopenia	With sarcopenia	*p*	Without sarcopenia	With sarcopenia	*p*
Sarcopenia defined as an appendicular lean body mass index cutoff point 5.6 for women and 7.5 for men and a handgrip z score < − 1						
* Number of participants*	*101*	*5*		*75*	*11*	
Total physical activity (MET-minutes/week) ^c^	742 (330–1589)^b^	495 (438–1836)	NS	1244 (693–2139)	594 (258–1836)	NS
Peak oxygen uptake (ml/min/kg) ^d^	14.4 ± 5.6^a^	7.0 ± 2.2	< 0.01	22.0 ± 8.3	15.6 ± 3.7	0.02
Peak respiratory exchange ratio (VCO_2_/VO_2_) ^d^	1.3 ± 0.1	1.4 ± 0.1	NS	1.3 ± 0.2	1.3 ± 0.2	NS
Peak workload (Watt) ^d^	93.2 ± 47.2	38.4 ± 9.8	0.01	171.1 ± 79.3	103.0 ± 56.5	< 0.01
Gross muscle efficiency (%)^d^	23.5 ± 7.7	23.0 ± 6.9	NS	23.0 ± 3.8	21.8 ± 6.7	NS
Physical activity level (Total/Resting energy expenditure) ^e^	1.47 ± 0.22	1.33 ± 0.12	NS	1.77 ± 0.38	1.57 ± 0.18	NS
Activity energy expenditure (Kcal/day) ^e^	445 (282–646)	221 (149–331)	0.03	926 (590–1533)	649 (447–706)	0.04
Sarcopenia defined as an appendicular lean body mass index cutoff point 5.6 for women and 7.5 for men						
* Number of participants*	*88*	*18*		*58*	*28*	
Total physical activity (MET-minutes/week)	731 (325–1570)^b^	808 (372–2319)	NS	1152 (678–2333)	1248 (454–1981)	NS
Peak oxygen uptake (ml/min/kg)	15.2 ± 5.5	8.8 ± 3.5	< 0.01	24.1 ± 7.7	15.1 ± 5.2	< 0.01
Peak respiratory exchange ratio (VCO_2_/VO_2_)	1.3 ± 0.1	1.3 ± 0.2	NS	1.3 ± 0.2	1.3 ± 0.2	NS
Peak workload (Watt)	100.5 ± 44.9	42.1 ± 25.2	< 0.01	194.6 ± 70.2	95.8 ± 53.3	< 0.01
Gross muscle efficiency (%)	24.1 ± 7.5	19.9 ± 7.2	0.03	23.5 ± 3.5	21.4 ± 5.3	0.03
Physical activity level (Total/Resting energy expenditure)	1.5 ± 0.2	1.4 ± 0.2	NS	1.8 ± 0.4	1.6 ± 0.3	NS
Activity energy expenditure (Kcal/day)	461 (285–742)	304 (221–416)	< 0.01	1008 (635–1619)	671 (541–811)	< 0.01
Sarcopenia defined as a z score for handgrip strength <= − 1						
* Number of participants*	*61*	*45*		*63*	*23*	
Total physical activity (MET-minutes/week)	742 (264–1928)	720 (400–1589)	NS	1547 (738–2346)	693 (330–1695)	< 0.01
Peak oxygen uptake (ml/min/kg)	12.7 ± 5.4	16.0 ± 5.6	< 0.01	22.6 ± 8.4	17.4 ± 6.2	< 0.01
Peak respiratory exchange ratio (VCO_2_/VO_2_)	1.3 ± 0.1	1.3 ± 0.1	NS	1.3 ± 0.1	1.3 ± 0.2	NS
Peak workload (Watt)	77.4 ± 45.0	108.4 ± 45.5	< 0.01	175.9 ± 78.6	125.4 ± 72.6	< 0.01
Gross muscle efficiency (%)	22.3 ± 8.4	24.9 ± 6.2	NS	23.3 ± 3.7	21.7 ± 5.4	NS
Physical activity level (Total/Resting energy expenditure)	1.4 ± 0.2	1.5 ± 0.3	NS	1.8 ± 0.4	1.6 ± 0.3	0.02
Activity energy expenditure (Kcal/day)	367 (250–555)	517 (295–754)	NS	951 (613–1619)	665 (448–943)	0.01

^a^= mean ± standard deviation ^b^ = median (interquartile range)

^c^= International physical activity questionnaire. ^d^= Exercise calorimetry. ^e^ = 72 h actigraph

**Fig. 1:**
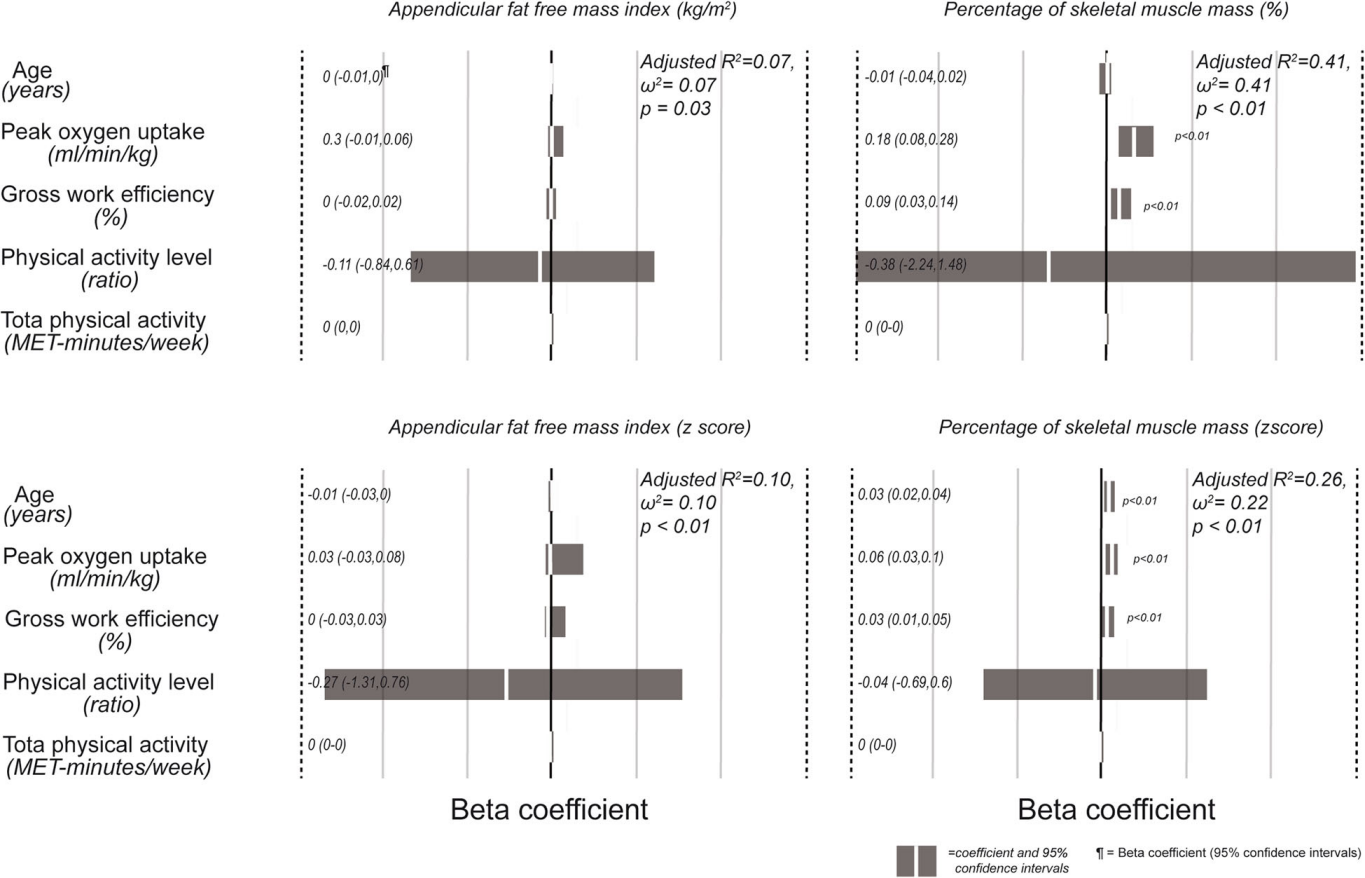
Beta coefficients of the multiple regression models for prediction of fat free mass in women, using as independent variables age, peak oxygen consumption, gross work efficiency, physical activity level and total physical activity derived from the International Physical Activity Questionnaire. A positive value indicates a direct association

**Fig. 2:**
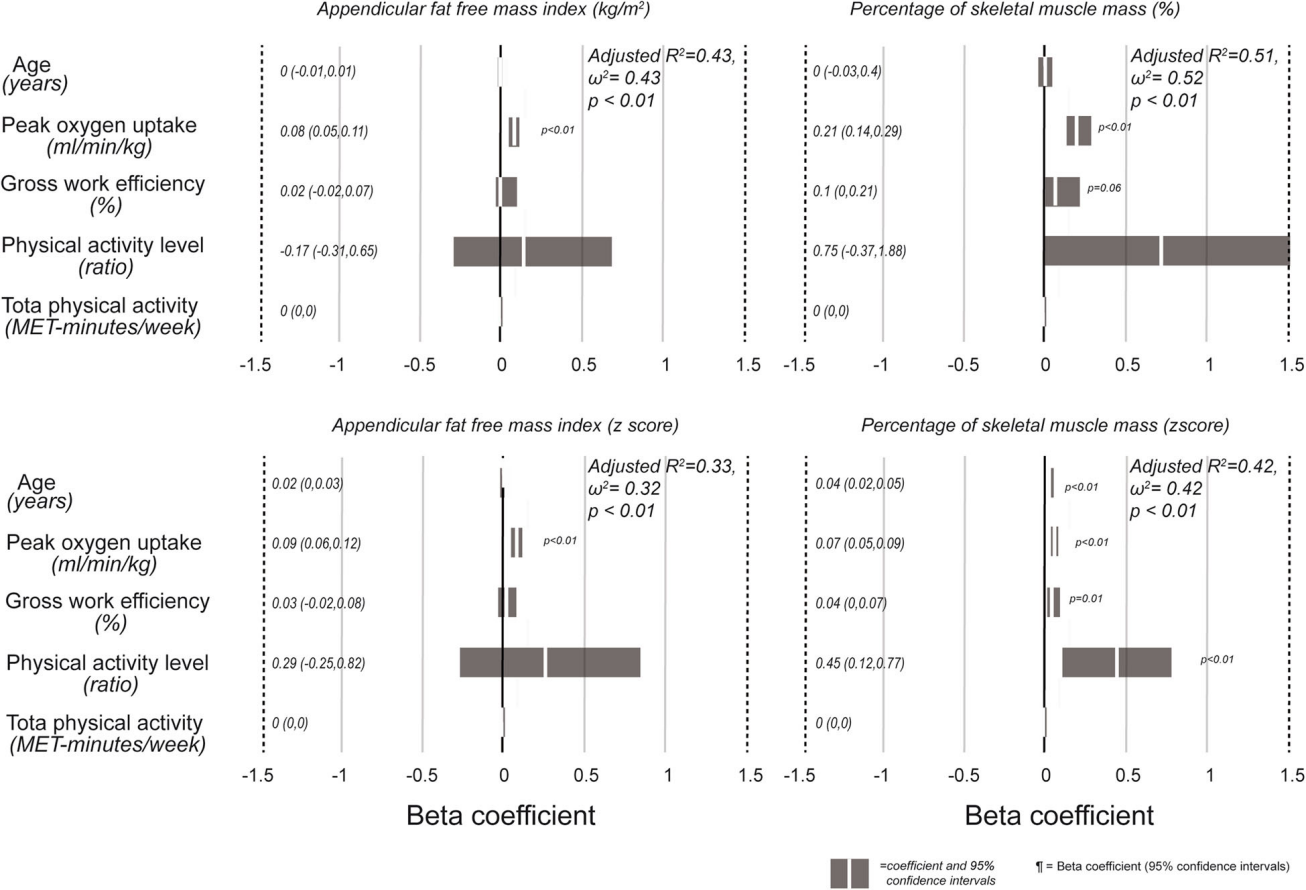
Beta coefficients of the multiple regression models for prediction of fat free mass in men, using as independent variables age, peak oxygen consumption, gross work efficiency, physical activity level and total physical activity derived from the International Physical Activity Questionnaire. A positive value indicates a direct association

Figures 1 and 2 show the regression models for appendicular fat free mass index and percentage of muscle mass, expressed as absolute values or z scores, for women and men. In general, the models had a higher r^2^ in men than women. In the former, peak oxygen consumption was always a significant and independent predictor of muscle mass. Among women, beta coefficients of peak oxygen consumption were only significant when fat free mass was expressed as percentage of skeletal muscle mass. Although the regressions are significant, the effect size of the models for men and women is low to intermediate, with r^2^ and ω^2^ values ranging from 0.07 to 0.52. In all models, except for the percentage of skeletal muscle mass expressed as z score in men, age was not a significant predictor of fat free mass.

**Fig. 3:**
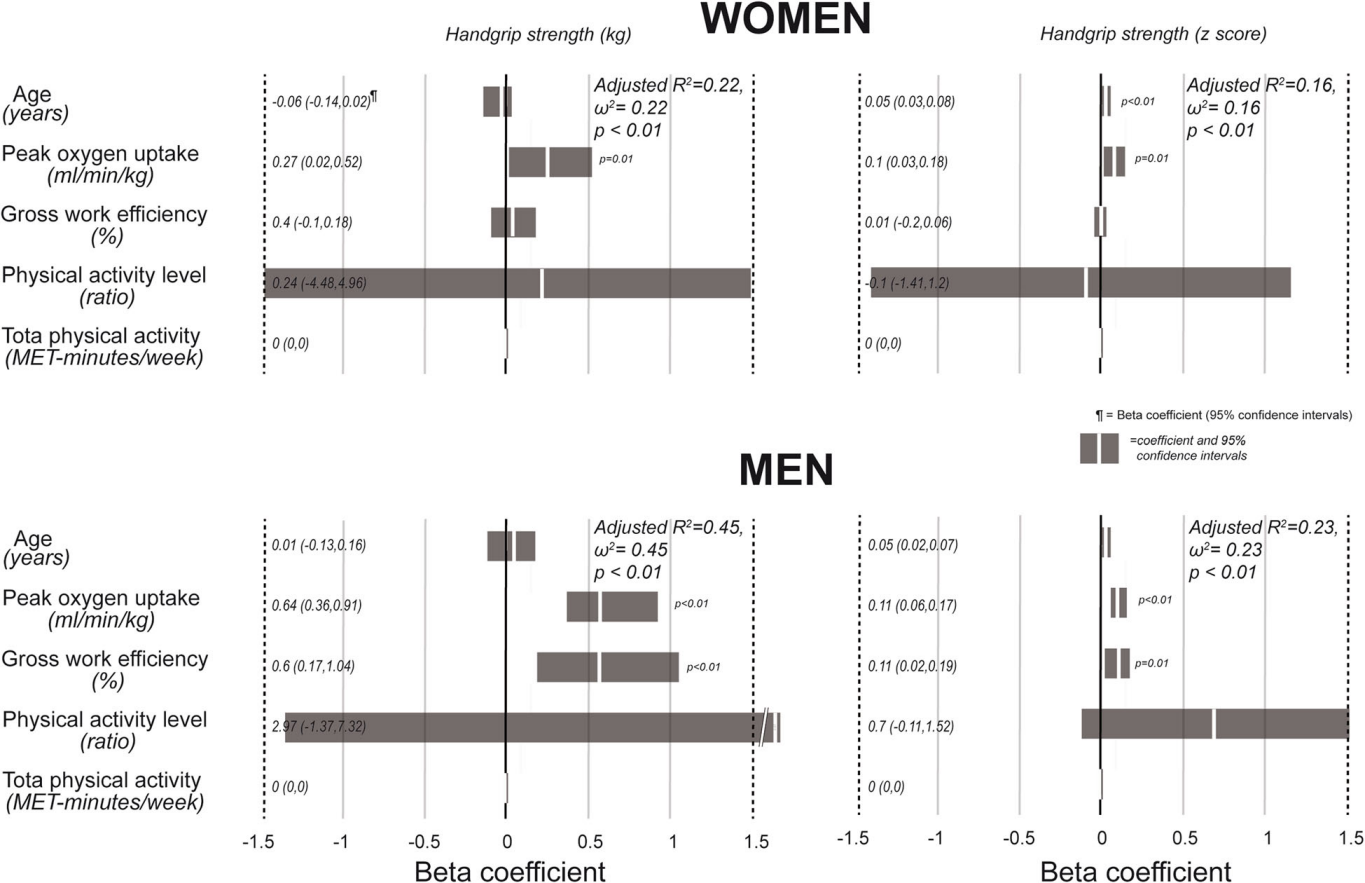
Beta coefficients of the multiple regression models for prediction of handgrip strength in men and women, using as independent variables age, peak oxygen consumption, gross work efficiency, physical activity level and total physical activity derived from the International Physical Activity Questionnaire. A positive value indicates a direct association

Figure 3 shows the results of the regression models when handgrip strength was the dependent variable. Again, the r^2^ values were higher in men and peak oxygen consumption was a significant and independent predictor in most models. Gross work efficiency was a significant predictor or handgrip strength in almost all models. Beta coefficients of physical activity level were only significant for the percentage of skeletal muscle mass expressed as z score in men. Total physical activity, calculated using the physical activity questionnaire did not have any predictive value. As in the case of fat free mass, the effect size of the models for men and women is low, with r^2^ and ω^2^ values ranging from 0.16 to 0.45.

## Discussion

We herein show that in young and old men and women, the presence of sarcopenia is associated with lower activity energy expenditure and a lower physical fitness, measured using an exercise calorimetry.

The definition of sarcopenia relies in the loss of muscle mass and function. We measured muscle mass using DEXA, which is accurate and reliable and accepted by most guidelines on sarcopenia [16]. Imaging methods such as magnetic resonance, ultrasound or computed tomography are also used, but their reliability is usually tested against DEXA [17]. The most widely used functional measure is handgrip strength, whose prognostic value is well established [18]. Therefore we used DEXA and handgrip strength to assess muscle mass and strength respectively. We have shown previously that both measures are predictive of mortality in our population of older subjects [19, 20]. The cutoff of muscle mass used to define sarcopenia was set at 1 standard deviation from the normal value, determined in a large set of participants of the same genetic and socioeconomic background reported previously [12]. In that report, we showed that using one instead of two standard deviations provides a better approach to determine sarcopenia in our population. The same cutoff was proposed by a Brazilian group of investigators previously [21]. The cutoff values for handgrip strength were derived from a similar group of participants, to avoid any regional bias in the determination of sarcopenia.

When comparing participants with and without sarcopenia defined using mass, strength or both, it is noteworthy that in all cases, subjects with sarcopenia had a lower peak oxygen uptake and activity energy expenditure, except in women, when sarcopenia was defined only by using handgrip strength. The differences in peak oxygen uptake have been seldom reported. The aforementioned Brazilian authors, performed an exercise test in a subgroup of participants with and without sarcopenic obesity finding differences in absolute but not relative peak oxygen uptake [21]. A Japanese group found an association between estimated oxygen uptake and skeletal muscle mass in men and women [22]. It is well known that training improves peak oxygen uptake, even in older people [23], but we are reporting results from participants who have not been trained. Thus, what we are observing are the effects of usual physical activity on this parameter [24], which is lower in participants with sarcopenia. There are several reports showing a lower activity energy expenditure in subjects with sarcopenia [25], therefore this finding is not surprising, but gives strength and reaffirms the differences found in peak oxygen uptake. On top of this, both peak oxygen consumption and usual physical activity are predictors of mortality healthy people [26, 27]. Thus, it is tempting to speculate if the prognostic value of sarcopenia on mortality is an independent effect or just a reflection of the higher level of sedentariness of people with this condition.

When muscle mass or strength were treated as a continuous variable in the multiple regression models, peak oxygen consumption was accepted as a significant predictor of fat free mass in men. In women the predictive capacity of this parameter was lower. These results reaffirm the concept that fitness is a protective factor against sarcopenia. The effect size of the regression equations was low, but they fulfill our purpose to show that physical fitness is related to the preservation of muscle mass across all ages. A report by Aggio et al. showed an association between sedentary time and the presence of severe sarcopenia, of a similar magnitude than the association herein reported [28]. A similar association was reported previously in older women. Surprisingly, the peak oxygen consumption values obtained by these authors were very similar to those obtained by us. However they interpreted the results, arguing that a lower muscle mass is the cause and not the consequence of a lower aerobic capacity [29]. Other authors also proposed this alternative hypothesis [30]. The only means of resolving is fitness or low muscle mass are the initiating event in the cascade of functional decline, is to perform longitudinal studies of cohorts, measuring the other factors that may affect aerobic capacity such as cardiovascular performance.

The association between handgrip strength and gross work efficiency is intuitively obvious, considering that both measures are a functional assessment of muscle. However, gross work efficiency and handgrip are assessing different muscular groups. This may be an indicator that a lower work efficiency is indicating an overall increase in the ATP cost for muscle contraction, phenomenon that is also associated with sarcopenia [31].

The International Physical Activity Questionnaire had no association with fat free mass. The lack of association of this parameter with peak oxygen uptake or activity energy expenditure, cast doubts about the accuracy of these types of questionnaires to objectively assess physical activity. Other authors report a significant albeit very low concordance between these type of questionnaires and data derived from accelerometry [32, 33]. Therefore, the interpretation of results obtained from these questionnaires should be very cautious and probably reserved only for large scale epidemiological studies. Using this questionnaire, we observed that young women had a lower total physical activity than the other participants. However this observation was not confirmed with Actigraphy, a much more accurate method.

The two main weaknesses of this study is that it is cross sectional and not having used doubly labeled water to assess activity energy expenditure. The latter is unsurmountable since the cost of doubly labeled water is too high. The former should encourage us to follow the participants of this cohort and perform a new assessment in the future to confirm is the actual loss of muscle mass, the essential aspect of sarcopenia, is associated with usual physical activity.

Our main strengths are that we studied both young and older people, showing that sarcopenia effectively occurs in young subjects and that physical activity is a risk factor for it in all age ranges. Also it is important to highlight that all comparisons were made separately by gender, avoiding the confounding effect of the striking differences in body composition and muscle strength between men and women [34]. It should also be pointed out that we used objective and accurate measures of physical activity such as accelerometry and cardiopulmonary exercise testing.

## Conclusions

Sedentariness is associated with sarcopenia in this group of participants and should be considered as an important risk factor for this condition.

## Abbreviations

AEE: Activity energy expenditure
DEXA: Double beam X ray absorptiometry
GE: General Electric
IPAQ: International Physical Activity Questionnaire
PAL: Physical activity level
TEE: Total energy expenditure
